# Acute Encephalitis Syndrome and Scrub Typhus in India

**DOI:** 10.3201/eid2308.162028

**Published:** 2017-08

**Authors:** Manoj V. Murhekar

**Affiliations:** National Institute of Epidemiology, Chennai, India

**Keywords:** acute encephalitis syndrome, scrub typhus, India, encephalitis, parasites, Orientia tsutsugamushi

**To the Editor:** I read with interest the article by Khan et al. (*1*). The National Vector-borne Disease Control Program reported >60,000 cases of acute encephalitis syndrome (AES) in India during 2010–2016; 8 states (Assam, Uttar Pradesh, West Bengal, Odisha, Tamil Nadu, Karnataka, Manipur, and Tripura) accounted for most cases (*2*). In many states, outbreaks of AES occur during the rainy season and are associated with high mortality rates. Following the national surveillance algorithm, AES cases are investigated for IgM against Japanese encephalitis, which accounted for <15% AES cases. Khan et al. reported 20% of AES cases were due to scrub typhus (*Orientia tsutsugamushi* infection) (*1*). In Gorakhpur, Uttar Pradesh, 62.7% of AES patients had *O. tsutsugamushi *IgM, with a case-fatality rate of 16.2% (*3*). Studies have reported central nervous system (CNS) involvement among a substantial number of scrub typhus patients from Dehradun, Uttarakhand; Vellore, Tamil Nadu; Puducherry; and Lucknow, Uttar Pradesh (*4**,**5*). Given these findings, managers of the national program should consider investigations for scrub typhus as part of the surveillance algorithm for AES cases.

Higher mortality rates among patients with AES could be attributed to delayed care. In Gorakhpur, where outbreaks of AES occur seasonally, the median interval between fever onset and hospitalization was 7 days (IQR 5–10 days) (*3*). Although intravenous azithromycin has been recommended for AES patients in Gorakhpur since 2014, fatality rates continue to be high, indicating low response to treatment after CNS involvement. Early treatment of patients with acute febrile illness with antimicrobial drugs such as doxycycline before CNS manifestations is critical. Assessing the contribution of scrub typhus among patients with acute febrile illness, developing algorithms for administering appropriate antimicrobial drugs, and educating healthcare providers about the use of doxycycline are crucial for reducing deaths among patients with AES in scrub typhus–endemic areas.
